# SIRT1/APE1 promotes the viability of gastric cancer cells by inhibiting p53 to suppress ferroptosis

**DOI:** 10.1515/med-2022-0620

**Published:** 2023-02-14

**Authors:** Huijin Zhao, Yuanyi Ding, Lan Zhang

**Affiliations:** Department of Gastroenterology, The Fourth Hospital of Hebei Medical University, Shijiazhuang City, Hebei Province, 050000, China; Department of No. 2 General Surgery, The Fourth Hospital of Hebei Medical University, Shijiazhuang City, Hebei Province, 050000, China

**Keywords:** SIRT1, APE1, p53, ferroptosis, gastric cancer

## Abstract

Gastric cancer (GC) is a common cancer worldwide with high mortality. Sirtuin 1 (SIRT1) and apurinic/apyrimidinic endodeoxyribonuclease 1 (APE1) are abnormally expressed in GC cells and related to p53, which is involved in ferroptosis. Thus, we explore the mechanism via which SIRT1, APE1, and p53 impact ferroptosis in GC cells. Specifically, GC cells were transfected with small-interfering RNA for SIRT1 (SiSIRT1) or small-interfering RNA for APE1 (SiAPE1) or with short-hairpin RNA for p53, and the cell viability, Fe^2+^, malondialdehyde (MDA), and glutathione (GSH) contents were detected by cell counting kit-8 assay and enzyme-linked immunosorbent assay. Western blot, immunofluorescence, and quantitative real-time polymerase chain reaction were conducted to quantify SIRT1, APE1, p53, solute carrier family 7 member 11 (SLC7A11), and glutathione peroxidase 4 (GPX4) levels in GC cells. Silencing of SIRT1 decreased viability, GSH content, and expressions of GPX4 and SLC7A11, while increased Fe^2+^, MDA content, and p53 expression in GC cells. Such aforementioned effects were reversed by APE1 overexpression. Also, SiAPE1 generated the same effects as SiSIRT1 on the above aspects, which was offset by p53 silencing. In short, SIRT1/APE1 promotes the growth of GC cells by targeting p53 to inhibit ferroptosis.

## 1 Introduction

Gastric cancer (GC) is a common malignancy all over the world with the fourth highest mortality rate [1]. In the initial stage, GC is mainly treated by surgery; however, patients after treatment still face risk of complications without obvious improvement [2]. Unfortunately, most patients are already in the late stage at first diagnosis, where the tumor metastases and chemotherapeutic resistance occur [3]. Recently, the programmed cell death ligand 1 and human epidermal growth factor receptor 2 have been proven to prolong the survival of patients with GC [4], signifying the feasibility of targeted therapy toward GC [5]. Thus, further research on the molecular mechanisms of GC is conducive to the development of targeted therapies for GC.

Ferroptosis is a form of non-apoptotic cell death characterized by iron-dependent lipid peroxidation [6]. Accumulating studies have demonstrated the involvement of ferroptosis in many cancers including GC [7,8,9]. Accordingly, ferroptosis has been recognized as a novel therapeutic target to eliminate cancer cells, which is not limited by chemotherapeutic resistance [10]. Previous studies have suggested that multiple pathways are implicated in the regulation of ferroptosis, including Nrf2, p53, and solute carrier family 7 member 11 (SLC7A11) [11]. It has been reported that p53 knockdown inhibited ferroptosis by regulating SLC7A11 and glutathione peroxidase 4 (GPX4) expressions in osteocytes [12]. In addition, Tanshinone IIA induces ferroptosis in GC cells through p53-mediated downregulation of SLC7A11 [13]. These studies indicated that p53 indeed mediated ferroptosis in cells. However, the mechanism via which ferroptosis participates in the development of GC cells needs further experiment.

Sirtuin 1 (SIRT1) is the founding member of class III histone deacetylases. In GC, SIRT1 is identified as a marker for prognosis and is involved in cell proliferation, cell cycle, autophagy, and drug resistance [14,15,16]. A previous study has demonstrated that SIRT1 is lowly expressed in GC following the silence of VEGF and causes the upregulation of p53 [17]. Ma et al. [18] also pointed out that SIRT1 could inhibit the ferroptosis-induced cell death through inhibiting the acetylation and protein levels of p53 in cardiomyocytes. Furthermore, SIRT1 could facilitate the development of cancer cells by deacetylating p53 [19]. Nonetheless, the function of SIRT1 and p53 in ferroptosis of GC has not been reported.

Moreover, it has been reported that SIRT1 silencing leads to the death of human embryonic stem cells via suppressing apurinic/apyrimidinic endodeoxyribonuclease 1 (APE1), a member of DNA repair enzymes [20]. APE1 is associated with tumorigenesis and indicates the poor prognosis of patients with GC [21]. Meanwhile, it is involved in DNA damage response and repair in GC [22]. In addition, APE1 participates in redox homeostasis, and its overexpression decreases the reactive oxygen species (ROS) content [23,24,25]. Of note, the ROS content is a main characteristic of ferroptosis [26]. Furthermore, APE1 is negatively regulated by p53, which is a regulatory factor in ferroptosis, and the interaction between these two boosts the degradation of p53 [27,28]. In colon cancer cells, the p53-mediated cell death is activated when the endonuclease activity of APE1 is retarded [29]. In GC cells, the APE1 expression is upregulated and APE1 silencing causes the cell death by inducing DNA damage [30,31]. DNA damage is repaired by p53, which, however, is hindered by the silencing of APE1 [32]. As such, APE1 may inhibit ferroptosis to prevent cell death [23]. It is worthy to fathom out the association between APE1 with SIRT1 or p53 in the ferroptosis in GC cells.

In light of this, an *in vitro* GC model was established using two GC cell lines in this study aiming to explore the regulatory network of SIRT1, APE1, and p53 in ferroptosis of GC cells.

## 2 Materials and methods

### 2.1 Cells and culture

Human GC cell lines including SNU-1 (CRL-5971) and AGS (CRL-1739) were acquired from the American Type Culture Collection (ATCC, Manassas, VA, USA). These GC cells, accordingly, were grown in the Rosewell Park Memorial Institute-1640 medium (30-2001, ATCC, USA) supplemented with 10% fetal bovine serum (S9020; Solarbio, Beijing, China) and 1% antibiotic/antifungal reagent (B1356-108, BIOEXPLORER Life Sciences, Boulder, CO, USA) at 37°C with 5% CO_2_. According to the cell growth conditions, the medium was changed every 2 days.

### 2.2 Cell transfection

The transfection was conducted using Lipofectamine^®^ 3000 (L3000008; Solarbio) according to the manufacturer’s instructions. The plasmid overexpressing APE1 was constructed with pcDNA vector (V38520; Thermo Fisher Scientific, Inc., Waltham, MA, USA). The empty pcDNA vector was used as a negative control (NC). Besides, the small-interfering RNA for SIRT1 (SiSIRT1, 5′-ATGGAGAAACATGTTATATATAC-3′), small-interfering RNA for APE1 (SiAPE1, 5′-CGGTATCGATAAGCTTGATATCG-3′), short-hairpin RNA for p53 (Shp53, 5′-CACCATCCACTACAACTACAT-3′), small-interfering RNA for NC (SiNC; A06001), and short-hairpin RNA for NC (ShNC; C03002) were designed and constructed by GenePharma (Shanghai, China).

Subsequently, SiNC, NC, SiSIRT1, SiAPE1, Shp53, and plasmid overexpressing APE1 were separately transfected into GC cells, while SiNC and NC, SiSIRT1 and plasmid overexpressing APE1, SiNC and ShNC, or SiAPE1 and Shp53 were co-transfected into other GC cells. After incubation for 48 h, these GC cells were collected for later experiments.

### 2.3 Quantitative real-time polymerase chain reaction (qRT-PCR)

Total RNA was extracted from GC cells with Total RNA Extractor (B511311; Sangon, Shanghai, China) and the purity was determined by NanoDrop™ One/OneC UV-Vis Spectrophotometer (701-058112; Thermo Fisher Scientific, Inc.). Then, 1 µg RNA was reversely transcribed into complementary DNA (cDNA) with Thermo Scientific RevertAid RT kit (K1691; Thermo Fisher Scientific, Inc.). Subsequently, the cDNAs were subjected to qRT-PCR with the specific primers of SIRT1, APE1, or p53 using Maxima SYBR Green/ROX qPCR (K0223, Thermo Fisher Scientific, Inc.) in the Mx3005P system (Agilent Technologies, Inc., CA, USA). The expressions of SIRT1, APE1, and p53 were calculated and determined using the 2^−ΔΔCt^ method with glyceraldehyde-3-phosphate dehydrogenase (GAPDH) as the normalization control [33]. The reaction conditions of PCR were as follows: 10 min at 95°C and then 40 cycles of 15 s at 94°C, 30 s at 58°C, and 15 s at 72°C. The sequences of primers are shown in Table 1.

**Table 1:** All primers in qRT-PCR experiments in this study

ID	Forward sequence (5′–3′)	Reverse sequence (5′–3′)
SIRT1	TAGCCTTGTCAGATAAGGAAGGA	ACAGCTTCACAGTCAACTTTGT
APE1	CCAGCCCTGTATGAGGACC	GGAGCTGACCAGTATTGATGAGA
p53	CAGCACATGACGGAGGTTGT	TCATCCAAATACTCCACACGC
GAPDH	GTCTCCTCTGACTTCAACAGCG	ACCACCCTGTTGCTGTAGCCAA

### 2.4 Cell counting kit-8 (CCK-8) assay

GC cells were collected at the logarithmic phase and resuspended in phosphate-buffered solution (PBS; P4474, Sigma-Aldrich, St. Louis, MO, USA). Subsequently, 100 µL of cell resuspension was added to 96-well plates and the density was adjusted to 1 × 10^3^ cells per well. Next, GC cells were transfected as aforementioned and cultured in a cell incubator (51033546, Thomas Scientific, Swedesboro, NJ, USA) at 37°C with 5% CO_2_ for 48 h. After that, 10 µL CCK-8 solution (C0037; Beyotime, Shanghai, China) was added to every well and incubated at 37°C for 4 h. The optical density (OD) value of every well was measured by an ELISA microplate reader (ELx808, Bio Tek, Winooski, VT, USA) at the wavelength of 450 nm.

### 2.5 Measurement of Fe^2+^ content

The Iron Assay Kit (MAK025; Sigma-Aldrich) was applied to determine the Fe^2+^ content in GC cells, AGS and SNU-1. In detail, GC cells (5 × 10^5^) were homogenized in Iron Assay Buffer and centrifuged (1,600 × *g*) at 4°C for 10 min, followed by the collection of supernatant. Then, 50 µL of supernatant was mixed with 5 µL of Iron Assay Buffer in 96-well plates and incubated at 25°C for 30 min in the dark. Thereafter, 100 µL of Iron Probe was added to every well and cultured at 25°C for 60 min avoiding light. Finally, the OD value was measured at the wavelength of 593 nm with an ELISA microplate reader.

### 2.6 Lipid peroxidation assay

The concentration of malondialdehyde (MDA) in GC cells was detected by Lipid Peroxidation (MDA) Assay Kit (ab118970; Abcam, UK). GC cells were first cultivated with thiobarbituric acid (TBA) solution at 95°C for 1 h and then lysed with RIPA lysis buffer (C500005; Sangon). After the mixture was centrifuged (13,000 × *g*) at 4°C for 5 min, the supernatant was collected. Next, the supernatant and the standard were chilled in ice bath for 10 min. The samples were finally transferred to a new 96-well microplate and the OD value was calculated using an microplate ELISA reader at the wavelength of 532 nm.

The glutathione (GSH) level in GC cells was determined by GSH ELISA Kit (D751008; Sangon). The GC cells were washed with cold PBS and digested with trypsin (C0202; Beyotime). Post centrifugation at 1,000 × *g* for 5 min, cells were collected and washed with PBS three times. The supernatant was collected for detection following the resuspension of cells with PBS and the centrifugation at 1,500 × *g* for 10 min. The 50 µL of standard and supernatant were separately added to the standard well and sample wells and mixed with 50 µL of working fluid (100 µg/mL) created by the mixture of standard and standard/sample diluent 1, followed by incubation for 45 min. After the incubation, the working fluid was removed, and 350 µL of washing solution was added to every well for 2 min. Thereafter, the samples were incubated with 100 µL of horseradish peroxidase (HRP)-conjugated streptavidin working solution at 37°C for 30 min. Next, 300 µL of washing solution was used to wash each well four times. After staining with 90 µL of chromogenic agent at 37°C for 15 min without light, 50 µL of stop solution was used to terminate the reaction and the OD value was measured by an ELISA microplate reader at the wavelength of 450 nm.

### 2.7 Immunofluorescence

GC cells at the logarithmic phase were collected and washed twice with PBS for 5 min. Then, cells were fixed with 4% paraformaldehyde (P1110; Solarbio) and washed three times with PBS for 5 min. Next, these cells were transparentized with Triton X-100 (A110694; Sangon) at room temperature for 10 min, rinsed with PBS, and incubated in an immunofluorescence blocking buffer (ab126587; Abcam) at room temperature for 30 min. Thereafter, cells were incubated with anti-GPX4 antibody (1 µg/mL, ab40993; Abcam) at 4°C overnight, followed by being washed three times with PBS for 5 min. Afterwards, cells were cultured with Goat Anti-Rabbit IgG H&L (Alexa Fluor^®^ 647) (1:1000, ab150083; Abcam) at room temperature for 1 h in the dark. Subsequently, cells were washed with PBS thrice for 5 min and stained with 4′,6-diamidino-2-phenylindole (E607303; Sangon), followed by being sealed with cover-glass (F518112; Sangon) away from light. The image was observed under a fluorescence microscope (Leica DM 6000B; Leica, Wetzlar, Germany) at the magnification of ×200.

### 2.8 Western blot

The proteins of cells were lysed in RIPA lysis buffer, and their concentrations were measured by Bicinchoninic Acid Assay (BCA) Protein Assay Kit (GK10009; Glpbio, Montclair, CA, USA). Following this, they were added to the protein loading buffer (C516031; Sangon) and heated at 95°C for 5 min to ensure the denaturation. Then, the proteins were separated by 12% sodium dodecyl sulfate polyacrylamide gel electrophoresis (P0678; Beyotime) and transferred onto polyvinylidene difluoride membranes (88585; Thermo Fisher Scientific, Inc.). Subsequently, the membranes were blocked with bovine serum albumin (37520; Thermo Fisher Scientific, Inc.) for 1 h and incubated with the primary antibodies against p53, SLC7A11, GPX4, or GAPDH (a loading control) at 4°C overnight. The expressions of proteins were normalized to that of GAPDH. After washing three times with tris-buffered saline wash buffer with Tween 20 (TBST; 28352; Thermo Fisher Scientific, Inc.) for 5 min, the proteins were incubated with secondary antibodies including goat anti-rabbit IgG H&L (HRP) and rabbit anti-mouse IgG H&L (HRP) for 1 h and washed three times with TBST for 5 min. The protein bands were visualized with High Sensitivity ECL Substrate Kit (ab133406; Abcam) and analyzed with an iBright™ CL1500 Imaging System (A44114; Thermo Fisher Scientific, Inc.). The information about all antibodies in this experiment is exhibited in Table 2.

**Table 2:** All antibodies information and sources in western blot in this study

ID	Catalog number	Company (country)	Molecular weight (kDa)	Dilution ratio/concentration
p53	ab26	Abcam (Cambridge, UK)	53	1 µg/mL
SLC7A11	ab175186	Abcam (Cambridge, UK)	55	1:1,000
GPX4	ab125066	Abcam (Cambridge, UK)	17	1:1,000
GAPDH	ab8245	Abcam (Cambridge, UK)	37	1:10,000
Goat anti-rabbit IgG H&L (HRP)	ab6702	Abcam (Cambridge, UK)		1:2,000
Rabbit anti-mouse IgG H&L (HRP)	ab6728	Abcam (Cambridge, UK)		1:2,000

### 2.9 Statistical analysis

All experiments in this study were repeated three times. All data were analyzed using GraphPad Prism 8.0 software (GraphPad, Inc., San Diego, CA, USA) and presented as mean ± standard deviation. The comparison among multiple groups was conducted by one-way analysis of variance. The data with *P* < 0.05 were defined to be statistically significant.

## 3 Results

### 3.1 APE1 overexpression reversed the effects of SIRT1 silencing on cell viability and contents of GSH, Fe^2+^, and MDA in SNU-1 and AGS cells

**Figure 1:**
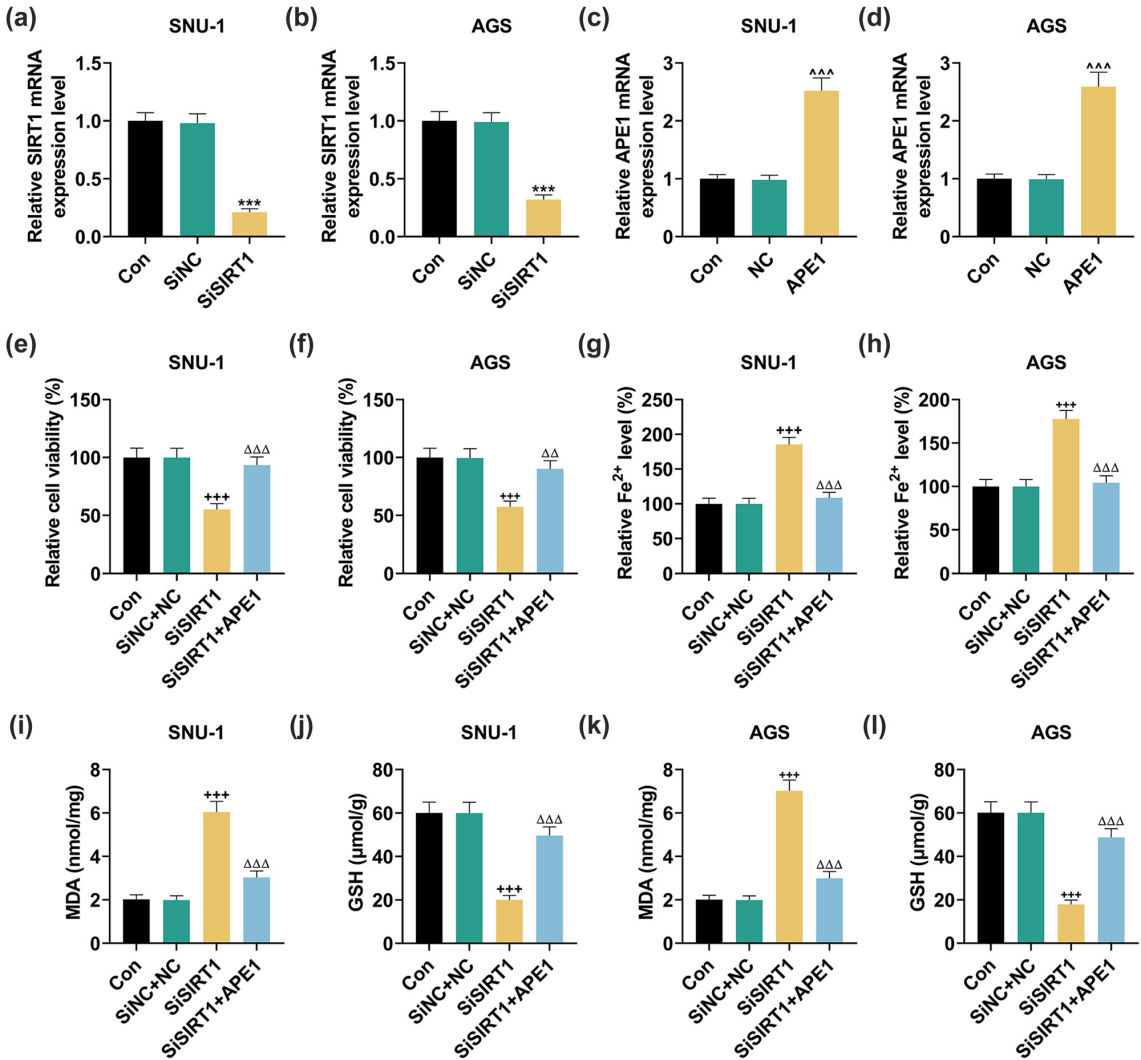
APE1 reversed the effects of SiSIRT1 on cell viability, Fe^2+^ content, and lipid peroxidation level in GC cells. (a–d) The expressions of SIRT1 and APE1 were determined by qRT-PCR in SNU-1 and AGS cells after the transfection of SiSIRT1 or APE1 overexpression plasmid. GAPDH was used as an internal reference. (e–l) The GC cells SNU-1 and AGS after transfection were separately divided into three groups: SiNC + NC, SiSIRT1, and SiSIRT1 + APE1. (e and f) The cell viability was detected by CCK-8 assay in SNU-1 and AGS cells with/without transfection. (g and h) The Iron Assay Kit was utilized to determine the Fe^2+^ content in SNU-1 and AGS cells following the transfection or not. (i–l) The MDA and GSH contents were detected in SNU-1 and AGS cells by Lipid Peroxidation (MDA) Assay Kit and GSH ELISA Kit, respectively, after the transfection or not. ^***^ *P* < 0.001 vs SiNC; ^^^^^ *P* < 0.001, vs NC; ^+++^ *P* < 0.001 vs SiNC + NC; ^ΔΔ^ *P* < 0.01, ^ΔΔΔ^ *P* < 0.001 vs SiSIRT1.

The relevant results of qRT-PCR are shown in Figure 1a–d. It was obvious that the SIRT1 expression was notably downregulated after the transfection of SiSIRT1 in both SNU-1 (Figure 1a, *P* < 0.001) and AGS (Figure 1b, *P* < 0.001) cells. The expression of APE1, contrarily, was clearly upregulated after the transfection of plasmid overexpressing APE1 in SNU-1 (Figure 1c, *P* < 0.001) and AGS (Figure 1d, *P* < 0.001) cells. These results indicated the successful transfection of SiSIRT1 and plasmid overexpressing APE1.

According to Figure 1e and f, the cell viability was significantly decreased after SIRT1 silencing in SNU-1 (Figure 1e, *P* < 0.001) and AGS (Figure 1f, *P* < 0.001) cells, the change of which was obviously offset by APE1 overexpression (Figure 1e and f, *P* < 0.01). Compared with the cells transfected with SiNC and NC, the memorably increased Fe^2+^ content was evident in SNU-1 (Figure 1g, *P* < 0.001) and AGS (Figure 1h, *P* < 0.001) cells transfected with SiSIRT1. Likewise, this impact of SiSIRT1 was neutralized by APE1 overexpression (Figure 1g and h, *P* < 0.001).

The graphical representation of GSH and MDA contents is shown in Figure 1i–l. Transfection of SiSIRT1 caused the overtly increased MDA content and the dramatically decreased GSH content in SNU-1 (Figure 1i and j, *P* < 0.001) and AGS (Figure 1k and l, *P* < 0.001) cells, the trend of which was offset by overexpressed APE1 (Figure 1i–l, *P* < 0.001). Collectively speaking, SIRT1 silencing decreased cell viability and GSH content but increased Fe^2+^ and MDA contents in SNU-1 and AGS cells. Such impacts, however, were reversed by APE1 overexpression.

### 3.2 APE1 overexpression reversed the effects of SIRT1 silencing on GPX4, SLC7A11, and p53 levels in SNU-1 and AGS cells

**Figure 2:**
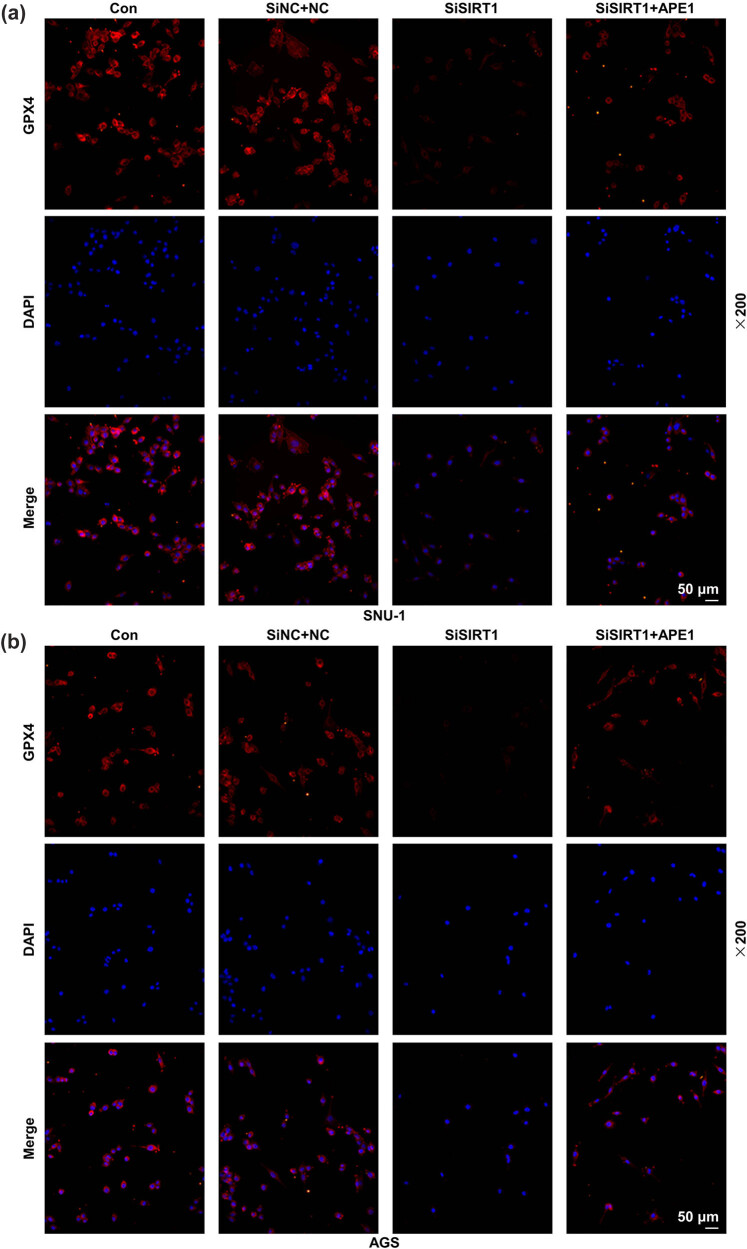
APE1 overexpression reversed the effect of SIRT1 silencing on GPX4 expression in GC cells. (a and b) The GC cells SNU-1 and AGS after transfection were separately divided into three groups: SiNC + NC, SiSIRT1, and SiSIRT1 + APE1. (a and b) The GPX4 expression was determined by immunofluorescence in GC cells after transfection (at a magnification of ×200, scale bar: 50 µm).

The results of immunofluorescence assay are presented in Figure 2a and b. It could be noticed that after SIRT1 silencing in SNU-1 and AGS cells, the GPX4 level was decreased, while such level was then elevated with the additional transfection of APE1 overexpression plasmid.

**Figure 3:**
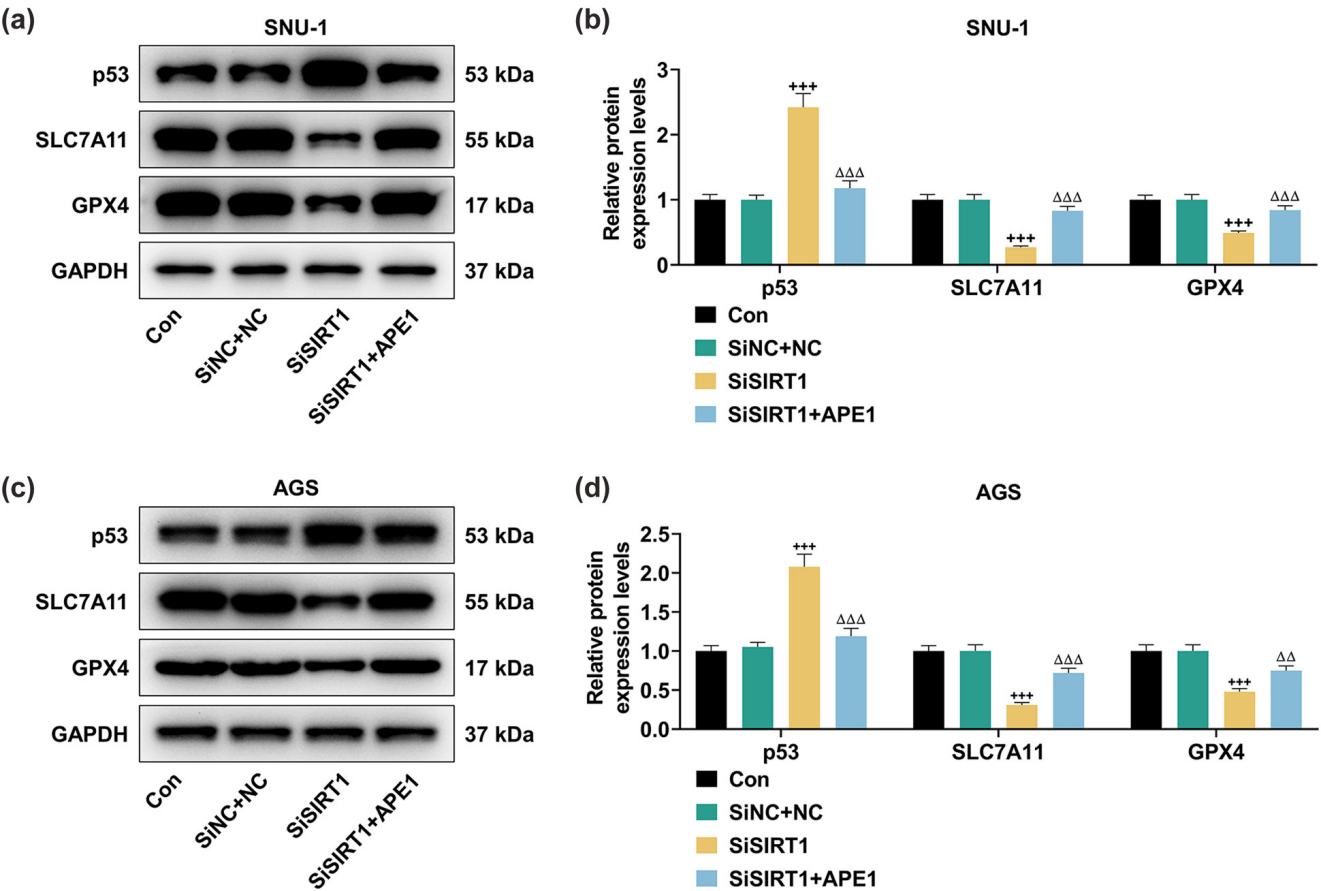
APE1 overexpression offset the effects of SIRT1 silencing on p53, SLC7A11, and GPX4 expressions in GC cells. (a–d) The SNU-1 and AGS cells after transfection were separately assigned into three groups: SiNC + NC, SiSIRT1, and SiSIRT1 + APE1. (a–d) The relevant results of Western blot showed the expressions of p53, SLC7A11, and GPX4 in GC cells after transfection. GAPDH was used as an internal reference.^+++^ *P* < 0.001 vs SiNC + NC; ^ΔΔΔ^ *P* < 0.001 vs SiSIRT1.

Based on the assay of Western blot, we found that following the transfection of SiSIRT1, the p53 protein level was remarkably augmented but SLC7A11 and GPX4 levels were signally lessened in SNU-1 (Figure 3a and b, *P* < 0.001) and AGS cells (Figure 3c and d, *P* < 0.001). Besides, in SNU-1 (Figure 3a and b, *P* < 0.001) and AGS (Figure 3c and d, *P* < 0.001) cells with the transfection of SiSIRT1, APE1 overexpression decreased p53 level but increased SLC7A11 and GPX4 levels. Taken together, the overexpression of APE1 reversed the effects of SIRT1 silencing on GPX4, SLC7A11, and p53 levels in SNU-1 and AGS cells.

### 3.3 p53 silencing offset the effects of APE1 depletion on cell viability and contents of GSH, Fe^2+^, and MDA in SNU-1 and AGS cells

**Figure 4:**
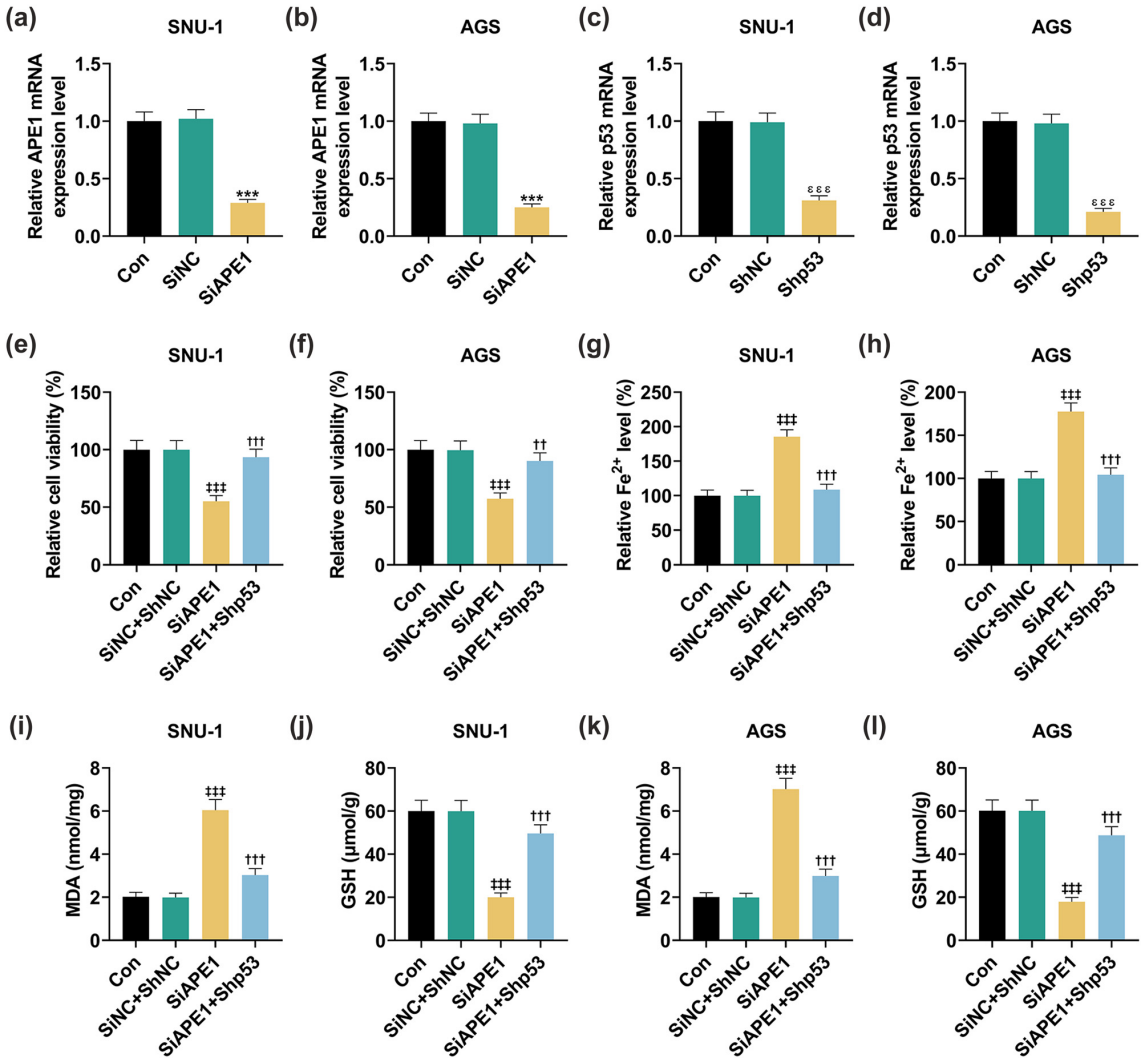
Shp53 neutralized the effects of SiAPE1 on cell viability, and contents of GSH, Fe^2+^, and MDA in GC cells. (a–d) The expressions of APE1 and p53 were determined by qRT-PCR in SNU-1 and AGS cells after the transfection with SiAPE1 or Shp53. GAPDH was used as an internal reference. (e–l) SNU-1 and AGS cells after transfection were separately distributed into three groups: SiNC + ShNC, SiAPE1, and SiAPE1 + Shp53. (e and f) The viability of SNU-1 and AGS cells after transfection or not was detected by CCK-8 assay. (g and h) The Iron Assay Kit was applied to determine the Fe^2+^ content in SNU-1 and AGS cells after transfection or not. (i–l) The MDA and GSH contents were detected by Lipid Peroxidation (MDA) Assay Kit and GSH ELISA Kit, respectively, in SNU-1 and AGS cells after transfection or not. ^***^ *P* < 0.001 vs SiNC; ^εεε^ *P* < 0.001 vs ShNC; ^‡‡‡^ *P* < 0.001 vs SiNC + ShNC; ^††^ *P* < 0.01, ^†††^ *P* < 0.001 vs SiAPE1.

The results of qRT-PCR (Figure 4a–d) demonstrated that the expression of APE1 was evidently downregulated after the transfection with SiAPE1 in SNU-1 (Figure 4a, *P* < 0.001) and AGS cells (Figure 4b, *P* < 0.001). Likewise, the expression of p53 was also markedly downregulated after transfection with Shp53 in SNU-1 (Figure 4c, *P* < 0.001) and AGS (Figure 4d, *P* < 0.001) cells.

In line with the data presented in Figure 4e and f, the cell viability was obviously suppressed after APE1 silencing in SUN-1 (Figure 4e, *P* < 0.001) and AGS (Figure 4f, *P* < 0.001) cells, but p53 silencing abrogated APE1 silencing-induced suppression of cell viability (Figure 4e and f, *P* < 0.01). Moreover, the transfection of SiAPE1 observably increased the Fe^2+^ content in SNU-1 (Figure 4g, *P* < 0.001) and AGS (Figure 4h, *P* < 0.001) cells; however, such effect was evidently offset after the transfection of Shp53 (Figure 4g and h, *P* < 0.001).

In addition, it was demonstrated that after APE1 silencing, the MDA content was memorably increased, while the GSH content was prominently decreased in SNU-1 (Figure 4i and j, *P* < 0.001) and AGS (Figure 4k and l, *P* < 0.001) cells. Such levels of MDA and GSH were pronouncedly reversed following the knockdown of p53 (Figure 4i–l, *P* < 0.001). In short, APE1 silencing decreased cell viability and GSH content yet increased Fe^2+^ and MDA contents in SNU-1 and AGS cells, while these effects were reversed by p53 silencing.

### 3.4 p53 silencing neutralized the effects of APE1 silencing on GPX4, SLC7A11, and p53 levels in SNU-1 and AGS cells

**Figure 5:**
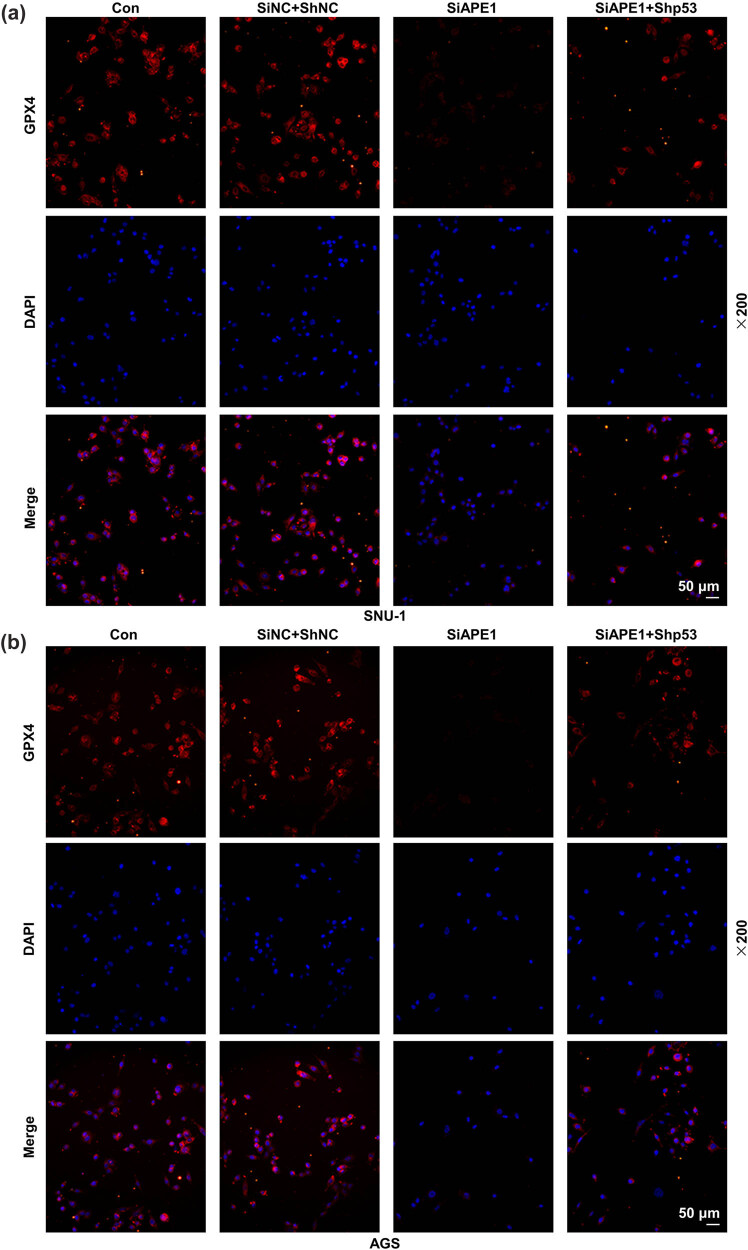
Shp53 reversed the effect of SiAPE1 on GPX4 expression in GC cells. (a and b) The SNU-1 and AGS cells after transfection were separately divided into three groups: SiNC + ShNC, SiAPE1, and SiAPE1 + Shp53. (a and b) The GPX4 expression was determined by immunofluorescence in transfected GC cells (at a magnification of ×200, scale bar: 50 µm).

As depicted in Figure 5a and b, GPX4 expression was downregulated after APE1 silencing in SNU-1 and AGS cells but was then restored by p53 silencing.

**Figure 6:**
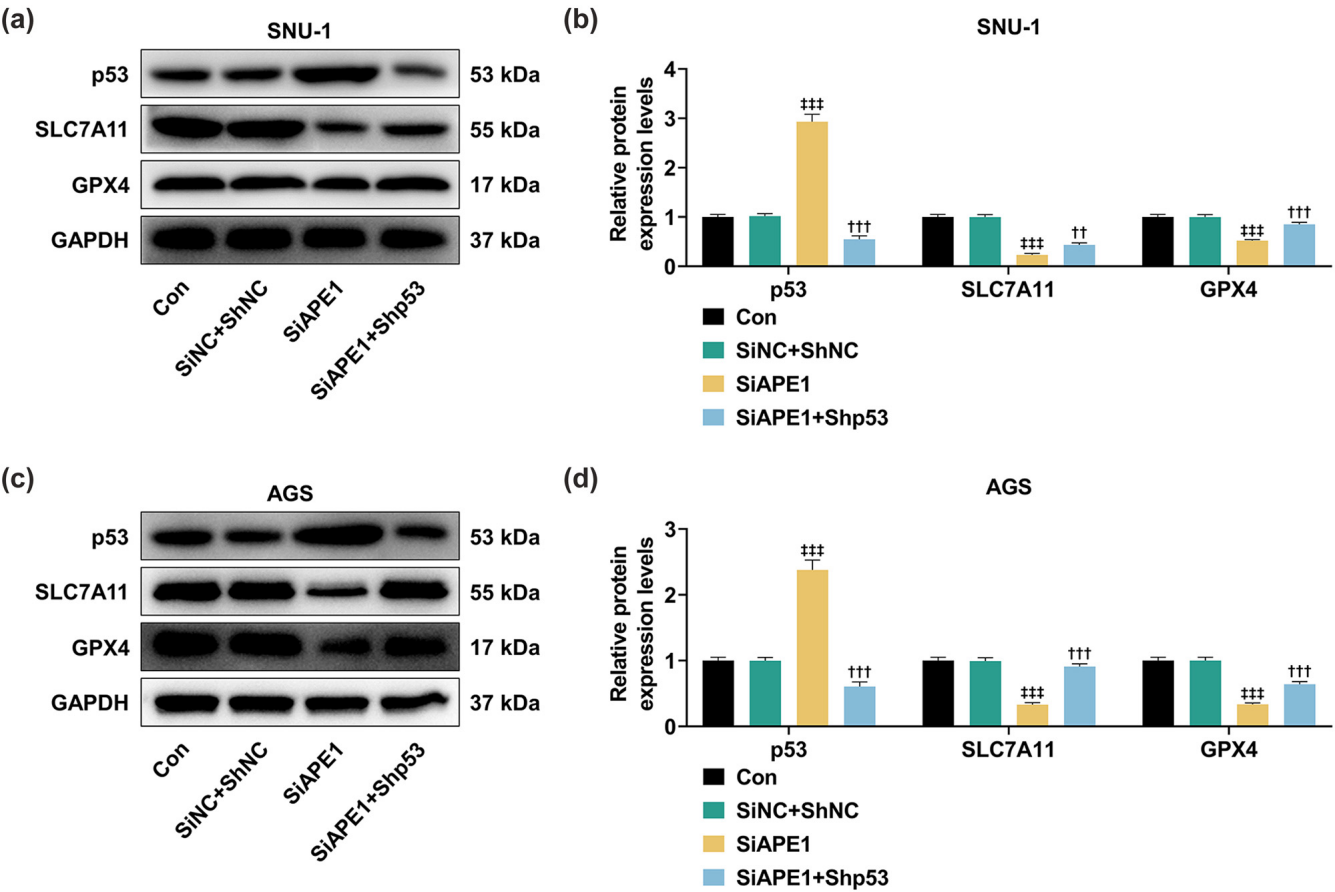
Shp53 offset the effects of SiAPE1 on p53, SLC7A11, and GPX4 expressions in GC cells. (a–d) The SNU-1 and AGS cells after transfection were separately divided into three groups: SiNC + ShNC, SiAPE1, and SiAPE1 + Shp53. (a–d) Western blot results showed the expressions of p53, SLC7A11, and GPX4 in GC cells after transfection. GAPDH was used as an internal reference. ^‡‡‡^ *P* < 0.001 vs SiNC + ShNC; ^††^ *P* < 0.01, ^†††^ *P* < 0.001 vs SiAPE1.

Based on the data (Figure 6a–d), after the silence of APE1, the p53 expression was notably promoted, while SLC7A11 and GPX4 expressions were prominently inhibited in SNU-1 (Figure 6a and b, *P* < 0.001) and AGS (Figure 6c and d, *P* < 0.001) cells. The changes in the expressions of these aforementioned proteins were significantly reversed by p53 silencing (Figure 6a–d, *P* < 0.01). In conclusion, APE1 silencing downregulated GPX4 and SLC7A11 levels yet upregulated p53 level in SNU-1 and AGS cells, the effects of which were offset by p53 silencing.

## 4 Discussion

In the present study, we found that SIRT1 silencing promoted p53 expression and ferroptosis yet inhibited the viability of GC cells, and APE1 overexpression reversed these effects of SIRT1 silencing on GC cells. Moreover, the depletion of APE1 had the similar effect to SIRT1 silencing in the GC cells, and the knockdown of p53 reversed the effects of APE1 silencing on GC cells. In conclusion, SIRT1/APE1 participates in the development of GC by targeting p53 to regulate ferroptosis.

Existing study has already demonstrated that SIRT1 is involved in the cell proliferation, apoptosis, and survival through regulating target gene expression and protein activities [34]. However, the function of SIRT1 in cancer is debatable [35]. It has been reported that the depletion of SIRT1 promotes the proliferation and migration of GC cells through signal transducer and activator of transcription (STAT3) signal pathway and nuclear factor-kappa B/Cyclin D1 [35,36]. Also, Deng et al. [37] found that miR-34a inhibits proliferation and boosts apoptosis of GC cells by inhibiting SIRT1. In addition, Hirai et al. [38] identified that the application of tenovin-6, a kind of SIRT1 inhibitor, causes GC cell death through activating death receptor 5, implying that the biological functions of SIRT1, including regulation of gene expression and DNA damage repair, are essential for the development of GC cells. In our study, we found that SIRT1 silencing caused the decrease in the viability of SNU-1 and AGS cells, which signified the role of SIRT1 as an oncogene and suggested that SIRT1 indeed played a role in modulating cell viability.

Besides, some other studies pointed out that SIRT1 is implicated in the inhibition of ferroptosis [39,40,41]. Su et al. [42] discovered that the ROS level is increased but GPX4 and SIRT1 expressions are decreased in ferric ammonium citrate (FAC)-induced THP-1 macrophages, the trends of which were reversed by SIRT1 overexpression. GPX4 is an inhibitor of ferroptosis [43,44], and its expression is positively regulated by the GSH content. Moreover, the depletion of GPX4 usually results in the onset of ferroptosis [45,46,47]. In addition, SLC7A11 is the other main regulator of ferroptosis like GPX4 and belongs to the system XC [48]. SLC7A11 has the capabilities of eliminating ROS and facilitating the GSH production so as to maintain the redox homeostasis and inhibit ferroptosis [49]. We uncovered that in GC cells with SIRT1 silencing, GSH content and GPX4 expression were diminished, while the Fe^2+^ content and the level of MDA, a marker of oxidative stress-induced lipid peroxidation [50], were all augmented. These findings thus reflected that SIRT1 silencing promoted the ferroptosis of GC cells. Furthermore, a previous study identified that p53 is activated by SIRT1 silencing and inhibits the level of SLC7A11 [51]. In this study, we confirmed that following the silence of SIRT1, the expressions of p53 [52] (a positive factor of ferroptosis) and SLC7A11 were upregulated and downregulated in GC cells, respectively, which further proved the positive effects of SIRT1 silencing in ferroptosis.

A previous study recognized that SIRT1 silencing decreases the expression of APE1 and causes the death of human embryonic stem cell [20]. Our results revealed that the effect of SIRT1 silencing on cell viability was reversed by APE1 overexpression, which proved the regulatory relation between SIRT1 and APE1. APE1 has also been evidenced to play a crucial role in ferroptosis due to its antioxidant property [23,53]. It has been reported that APE1 overexpression decreases the accumulation of ROS and increases the GSH content in myocardial ischemia–reperfusion-induced cardiomyocytes [54]. The relation between APE1 with GPX4 or SLC7A11 has not been reported, but previous studies have recognized that APE1 and GPX4 are regulated by SIRT1 in cardiomyocytes and kidney [18,55]. In our study, when compared with those in cells transfected with SiSIRT1 alone, cell viability, GSH content, GPX4 level, and SLC7A11 level were increased but p53 level, Fe^2+^ content, and MDA content were decreased in cells co-transfected with SiSIRT1 and APE1 overexpression plasmid. It could be then concluded that APE1 silencing inhibited cell viability and promoted ferroptosis, while SIRT1 silencing promoted ferroptosis by inhibiting APE1 expression.

APE1 is confirmed to be lowly expressed in GC cells [30] and be closely associated with p53 [56,57]. The negative regulatory network between APE1 and p53 has been already proved in lung endothelium [28]. Zhu et al. found that the interaction of APE1 and p53 promotes the degradation of p53 in other cancer cells like non-small-lung cancer cells and cervical cancer cells [57]. However, the specific relationship between APE1 and p53 in GC cells remains to be clarified. Our discoveries revealed that p53 silencing elevated the cell viability, GPX4 expression, and SLC7A11 expression, while diminishing the Fe^2+^ content, MDA content, and p53 expression in GC cells transfected with ShAPE1. These discoveries indicated that APE1 might inhibit ferroptosis via inactivating p53-mediated signal pathway.

## 5 Conclusions

Collectively speaking, we identify that SIRT1 and APE1 can regulate ferroptosis in GC cells and affect the development of GC cells by targeting p53. However, it should be noticed that there may exist some other signal pathways and factors of ferroptosis involved. Accordingly, the regulatory network targeting ferroptosis in GC cells needs to be validated in further experiments.

## References

[1] Sung H, Ferlay J, Siegel RL, Laversanne M, Soerjomataram I, Jemal A, et al. Global cancer statistics 2020: GLOBOCAN estimates of incidence and mortality worldwide for 36 cancers in 185 countries. CA Cancer J Clin. 2021;71(3):209–49. 10.3322/caac.21660 33538338

[2] Cha JH, Jang JS. Correlation between healing type of lesion and recurrence in gastric neoplastic lesions after endoscopic submucosal dissection. Turk J Gastroenterol. 2020;31(1):36–41. 10.5152/tjg.2020.18764 PMC7075678 32009612

[3] Sexton RE, Al Hallak MN, Diab M, Azmi AS. Gastric cancer: A comprehensive review of current and future treatment strategies. Cancer Metastasis Rev. 2020;39(4):1179–203. 10.1007/s10555-020-09925-3 PMC7680370 32894370

[4] Joshi SS, Badgwell BD. Current treatment and recent progress in gastric cancer. CA Cancer J Clin. 2021;71(3):264–79. 10.3322/caac.21657 PMC9927927 33592120

[5] Patel TH, Cecchini M. Targeted therapies in advanced gastric cancer. Curr Treat Options Oncol. 2020;21(9):70. 10.1007/s11864-020-00774-4 32725377

[6] Fu D, Wang C, Yu L, Yu R. Induction of ferroptosis by ATF3 elevation alleviates cisplatin resistance in gastric cancer by restraining Nrf2/Keap1/xCT signaling. Cell Mol Biol Lett. 2021;26(1):26. 10.1186/s11658-021-00271-y PMC8186082 34098867

[7] Ni H, Qin H, Sun C, Liu Y, Ruan G, Guo Q, et al. MiR-375 reduces the stemness of gastric cancer cells through triggering ferroptosis. Stem Cell Res Ther. 2021;12(1):325. 10.1186/s13287-021-02394-7 PMC8180146 34090492

[8] Yao MY, Liu T, Zhang L, Wang MJ, Yang Y, Gao J. Role of ferroptosis in neurological diseases. Neurosci Lett. 2021;747:135614. 10.1016/j.neulet.2020.135614 33485988

[9] Chen X, Kang R, Kroemer G, Tang D. Targeting ferroptosis in pancreatic cancer: A double-edged sword. Trends Cancer. 2021;7(10):891–901. 10.1016/j.trecan.2021.04.005 34023326

[10] Hassannia B, Vandenabeele P, Vanden Berghe T. Targeting ferroptosis to iron out cancer. Cancer Cell. 2019;35(6):830–49. 10.1016/j.ccell.2019.04.002 31105042

[11] Koppula P, Zhuang L, Gan B. Cystine transporter SLC7A11/xCT in cancer: Ferroptosis, nutrient dependency, and cancer therapy. Protein cell. 2021;12(8):599–620. 10.1007/s13238-020-00789-5 PMC8310547 33000412

[12] Sun F, Zhou JL, Liu ZL, Jiang ZW, Peng H. Dexamethasone induces ferroptosis via P53/SLC7A11/GPX4 pathway in glucocorticoid-induced osteonecrosis of the femoral head. Biochem Biophys Res Commun. 2022;602:149–55. 10.1016/j.bbrc.2022.02.112 35276555

[13] Guan Z, Chen J, Li X, Dong N. Tanshinone IIA induces ferroptosis in gastric cancer cells through p53-mediated SLC7A11 down-regulation. Bioscience reports. 2020;40:8. 10.1042/BSR20201807 PMC7953492 32776119

[14] Qiu G, Li X, Che X, Wei C, He S, Lu J, et al. SIRT1 is a regulator of autophagy: Implications in gastric cancer progression and treatment. FEBS letters. 2015;589(16):2034–42. 10.1016/j.febslet.2015.05.042 26049033

[15] Liu H, Liu N, Zhao Y, Zhu X, Wang C, Liu Q, et al. Oncogenic USP22 supports gastric cancer growth and metastasis by activating c-Myc/NAMPT/SIRT1-dependent FOXO1 and YAP signaling. Aging. 2019;11(21):9643–60. 10.18632/aging.102410 PMC6874452 31689236

[16] Zhang W, Liao K, Liu D. MiRNA-12129 suppresses cell proliferation and block cell cycle progression by targeting SIRT1 in GASTRIC cancer. Technology in Cancer research & Treatment. 2020;19:1533033820928144. 10.1177/1533033820928144 PMC7281879 32508267

[17] Sun P, Yu H, Zhang WQ, Hu M, Lv R. Lentivirus-mediated siRNA targeting VEGF inhibits gastric cancer growth in vivo. Oncol Rep. 2012;28(5):1687–92. 10.3892/or.2012.1966 22895814

[18] Ma S, Sun L, Wu W, Wu J, Sun Z, Ren J. USP22 protects against myocardial ischemia-reperfusion injury via the SIRT1-p53/SLC7A11-dependent inhibition of ferroptosis-induced cardiomyocyte death. Front Physiol. 2020;11:551318. 10.3389/fphys.2020.551318 PMC7609439 33192549

[19] Lin XL, Li K, Yang Z, Chen B, Zhang T. Dulcitol suppresses proliferation and migration of hepatocellular carcinoma via regulating SIRT1/p53 pathway. Phytomedicine. 2020;66:153112. 10.1016/j.phymed.2019.153112 31786318

[20] Jang J, Huh YJ, Cho HJ, Lee B, Park J, Hwang DY, et al. SIRT1 enhances the survival of human embryonic stem cells by promoting DNA repair. Stem Cell Reports. 2017;9(2):629–41. 10.1016/j.stemcr.2017.06.001 PMC5549766 28689995

[21] Qing Y, Li Q, Ren T, Xia W, Peng Y, Liu GL, et al. Upregulation of PD-L1 and APE1 is associated with tumorigenesis and poor prognosis of gastric cancer. Drug Des Dev Ther. 2015;9:901–9. 10.2147/DDDT.S75152 PMC4338255 25733810

[22] Manoel-Caetano FS, Rossi AFT, Calvet de Morais G, Severino FE, Silva AE. Upregulation of the APE1 and H2AX genes and miRNAs involved in DNA damage response and repair in gastric cancer. Genes Diseases. 2019;6(2):176–84. 10.1016/j.gendis.2019.03.007 PMC6545450 31194025

[23] Guo N, Chen Y, Zhang Y, Deng Y, Zeng F, Li X. Potential role of APEX1 during ferroptosis. Front Oncol. 2022;12:798304. 10.3389/fonc.2022.798304 PMC8927806 35311089

[24] den Hartog G, Chattopadhyay R, Ablack A, Hall EH, Butcher LD, Bhattacharyya A, et al. Regulation of rac1 and reactive oxygen species production in response to infection of gastrointestinal epithelia. PLoS Pathog. 2016;12(1):e1005382. 10.1371/journal.ppat.1005382 PMC4711900 26761793

[25] Kang B, Mu S, Yang Q, Guo S, Chen X, Guo H. Ape1 protects against MPP + -induced neurotoxicity through ERK1/2 signaling in PC12 cells. Neuroreport. 2017;28(1):10–6. 10.1097/WNR.0000000000000712 27893608

[26] Li J, Cao F, Yin HL, Huang ZJ, Lin ZT, Mao N, et al. Ferroptosis: Past, present and future. Cell Death Dis. 2020;11(2):88. 10.1038/s41419-020-2298-2 PMC6997353 32015325

[27] Tarangelo A, Magtanong L, Bieging-Rolett KT, Li Y, Ye J, Attardi LD, et al. p53 suppresses metabolic stress-induced ferroptosis in cancer cells. Cell Rep. 2018;22(3):569–75. 10.1016/j.celrep.2017.12.077 PMC5791910 29346757

[28] Uddin MA, Akhter MS, Siejka A, Catravas JD, Barabutis N. P53 supports endothelial barrier function via APE1/Ref1 suppression. Immunobiology. 2019;224(4):532–8. 10.1016/j.imbio.2019.04.008 PMC6682453 31023490

[29] McNeill DR, Narayana A, Wong HK, Wilson 3rd DM. Inhibition of Ape1 nuclease activity by lead, iron, and cadmium. Environ Health Perspect. 2004;112(7):799–804. 10.1289/ehp.7038 PMC1241995 15159209

[30] Ajucarmelprecilla A, Pandi J, Dhandapani R, Ramanathan S, Chinnappan J, Paramasivam R, et al. In Silico identification of hub genes as observing biomarkers for gastric cancer metastasis. Evidence Based Complementary Altern Med. 2022;2022:6316158. 10.1155/2022/6316158 PMC9078768 35535159

[31] Manoel-Caetano FS, Rossi AFT, Ribeiro ML, Prates J, Oliani SM, Silva AE. Hydrogen peroxide and Helicobacter pylori extract treatment combined with APE1 knockdown induce DNA damage. G2/M arrest and cell death in gastric cancer cell line. DNA Repair (Amst). 2020;96:102976. 10.1016/j.dnarep.2020.102976 33065487

[32] Cun Y, Dai N, Li M, Xiong C, Zhang Q, Sui J, et al. APE1/Ref-1 enhances DNA binding activity of mutant p53 in a redox-dependent manner. Oncol Rep. 2014;31(2):901–9. 10.3892/or.2013.2892 24297337

[33] Livak KJ, Schmittgen TD. Analysis of relative gene expression data using real-time quantitative PCR and the 2(-Delta Delta C(T)) method. Methods. 2001;25(4):402–8. 10.1006/meth.2001.1262 11846609

[34] Yuan H, Su L, Chen WY. The emerging and diverse roles of sirtuins in cancer: A clinical perspective. Onco Targets Ther. 2013;6:1399–416. 10.2147/OTT.S37750 PMC3797239 24133372

[35] Yang Q, Wang B, Gao W, Huang S, Liu Z, Li W, et al. SIRT1 is downregulated in gastric cancer and leads to G1-phase arrest via NF-kappaB/Cyclin D1 signaling. Mol Cancer Res. 2013;11(12):1497–507. 10.1158/1541-7786.MCR-13-0214 24107295

[36] Zhang S, Yang Y, Huang S, Deng C, Zhou S, Yang J, et al. SIRT1 inhibits gastric cancer proliferation and metastasis via STAT3/MMP-13 signaling. J Cell Physiol. 2019;234(9):15395–406. 10.1002/jcp.28186 30710340

[37] Deng X, Zheng H, Li D, Xue Y, Wang Q, Yan S, et al. MicroRNA-34a regulates proliferation and apoptosis of gastric cancer cells by targeting silent information regulator 1. Exp Ther Med. 2018;15(4):3705–14. 10.3892/etm.2018.5920 PMC5863600 29581731

[38] Hirai S, Endo S, Saito R, Hirose M, Ueno T, Suzuki H, et al. Antitumor effects of a sirtuin inhibitor, tenovin-6, against gastric cancer cells via death receptor 5 up-regulation. PLoS One. 2014;9(7):e102831. 10.1371/journal.pone.0102831 PMC4102575 25033286

[39] Dang R, Wang M, Li X, Wang H, Liu L, Wu Q, et al. Edaravone ameliorates depressive and anxiety-like behaviors via Sirt1/Nrf2/HO-1/Gpx4 pathway. J Neuroinflammation. 2022;19(1):41. 10.1186/s12974-022-02400-6 PMC8822843 35130906

[40] Qiongyue Z, Xin Y, Meng P, Sulin M, Yanlin W, Xinyi L, et al. Post-treatment with irisin attenuates acute kidney injury in sepsis mice through anti-ferroptosis via the SIRT1/Nrf2 pathway. Front Pharmacol. 2022;13:857067. 10.3389/fphar.2022.857067 PMC8970707 35370723

[41] Wang C, Liu T, Tong Y, Cui R, Qu K, Liu C, et al. Ulinastatin protects against acetaminophen-induced liver injury by alleviating ferroptosis via the SIRT1/NRF2/HO-1 pathway. Am J Transl Res. 2021;13(6):6031–42. PMC8290678 34306342

[42] Su G, Yang W, Wang S, Geng C, Guan X. SIRT1-autophagy axis inhibits excess iron-induced ferroptosis of foam cells and subsequently increases IL-1Beta and IL-18. Biochem Biophys Res Commun. 2021;561:33–9. 10.1016/j.bbrc.2021.05.011 34000515

[43] Doll S, Freitas FP, Shah R, Aldrovandi M, da Silva MC, Ingold I, et al. FSP1 is a glutathione-independent ferroptosis suppressor. Nature. 2019;575(7784):693–8. 10.1038/s41586-019-1707-0 31634899

[44] Mao C, Liu X, Zhang Y, Lei G, Yan Y, Lee H, et al. DHODH-mediated ferroptosis defence is a targetable vulnerability in cancer. Nature. 2021;593(7860):586–90. 10.1038/s41586-021-03539-7 PMC8895686 33981038

[45] Jelinek A, Heyder L, Daude M, Plessner M, Krippner S, Grosse R, et al. Mitochondrial rescue prevents glutathione peroxidase-dependent ferroptosis. Free Radic Biol Med. 2018;117:45–57. 10.1016/j.freeradbiomed.2018.01.019 29378335

[46] Zhang Y, Lu X, Tai B, Li W, Li T. Ferroptosis and its multifaceted roles in cerebral stroke. Front Cell Neurosci. 2021;15:615372. 10.3389/fncel.2021.615372 PMC8209298 34149358

[47] Kitakata H, Endo J, Matsushima H, Yamamoto S, Ikura H, Hirai A, et al. MITOL/MARCH5 determines the susceptibility of cardiomyocytes to doxorubicin-induced ferroptosis by regulating GSH homeostasis. J Mol Cell Cardiol. 2021;161:116–29. 10.1016/j.yjmcc.2021.08.006 34390730

[48] Lei G, Zhang Y, Koppula P, Liu X, Zhang J, Lin SH, et al. The role of ferroptosis in ionizing radiation-induced cell death and tumor suppression. Cell Res. 2020;30(2):146–62. 10.1038/s41422-019-0263-3 PMC7015061 31949285

[49] Hu P, Xu Y, Jiang Y, Huang J, Liu Y, Wang D, et al. The mechanism of the imbalance between proliferation and ferroptosis in pulmonary artery smooth muscle cells based on the activation of SLC7A11. Eur J Pharmacol. 2022;928:175093. 10.1016/j.ejphar.2022.175093 35700835

[50] Park MW, Cha HW, Kim J, Kim JH, Yang H, Yoon S, et al. NOX4 promotes ferroptosis of astrocytes by oxidative stress-induced lipid peroxidation via the impairment of mitochondrial metabolism in Alzheimer’s diseases. Redox Biol. 2021;41:101947. 10.1016/j.redox.2021.101947 PMC8027773 33774476

[51] Xie Y, Zhu S, Song X, Sun X, Fan Y, Liu J, et al. The tumor suppressor p53 limits ferroptosis by blocking DPP4 activity. Cell Rep. 2017;20(7):1692–704. 10.1016/j.celrep.2017.07.055 28813679

[52] Jiang L, Kon N, Li T, Wang SJ, Su T, Hibshoosh H, et al. Ferroptosis as a p53-mediated activity during tumour suppression. Nature. 2015;520(7545):57–62. 10.1038/nature14344 PMC4455927 25799988

[53] Sriramajayam K, Peng D, Lu H, Zhou S, Bhat N, McDonald OG, et al. Activation of NRF2 by APE1/REF1 is redox-dependent in Barrett’s related esophageal adenocarcinoma cells. Redox Biol. 2021;43:101970. 10.1016/j.redox.2021.101970 PMC8082268 33887608

[54] Hao J, Du H, Liu F, Lu JC, Yang XC, Cui W. Apurinic/apyrimidinic endonuclease/redox factor 1 (APE1) alleviates myocardial hypoxia-reoxygenation injury by inhibiting oxidative stress and ameliorating mitochondrial dysfunction. Exp Ther Med. 2019;17(3):2143–51. 10.3892/etm.2019.7212 PMC6395998 30867702

[55] Fang D, Wang Y, Zhang Z, Yang D, Gu D, He B, et al. Calorie restriction protects against contrast-induced nephropathy via SIRT1/GPX4 activation. Oxid Med Cell Longev. 2021;2021:2999296. 10.1155/2021/2999296 PMC8548166 34712381

[56] Codrich M, Comelli M, Malfatti MC, Mio C, Ayyildiz D, Zhang C, et al. Inhibition of APE1-endonuclease activity affects cell metabolism in colon cancer cells via a p53-dependent pathway. DNA Repair. 2019;82:102675. 10.1016/j.dnarep.2019.102675 PMC7092503 31450087

[57] Zhu J, Zhang C, Qing Y, Cheng Y, Jiang X, Li M, et al. Genistein induces apoptosis by stabilizing intracellular p53 protein through an APE1-mediated pathway. Free Radic Biol Med. 2015;86:209–18. 10.1016/j.freeradbiomed.2015.05.030 26032169

